# Uptake of Seeds Secondary Metabolites by *Virola surinamensis* Seedlings

**DOI:** 10.1155/2012/721494

**Published:** 2012-03-18

**Authors:** Massuo Jorge Kato, Massayoshi Yoshida, Norberto Peporine Lopes, Denise Brentan da Silva, Alberto José Cavalheiro

**Affiliations:** ^1^Instituto de Química, Universidade de São Paulo, CP 26077, 05599-970 São Paulo, SP, Brazil; ^2^Núcleo de Pesquisa em Produtos Naturais e Sintéticos (NPPNS), Faculdade de Ciências Farmacêuticas de Ribeirão Preto, Universidade de São Paulo, 14040-903 Ribeirão Preto, SP, Brazil; ^3^Lychnoflora Pesquisa e Desenvolvimento em Produtos Naturais LTDA, Incubadora Supera, Campus da USP, 14040-900 Ribeirão Preto, SP, Brazil; ^4^Núcleo de Bioensaio, Biossíntese e Ecofisiologia de Produtos Naturais (NuBBE), Instituto de Química, Universidade Estadual Paulista, CP 355, 14800-900 Araraquara, SP, Brazil

## Abstract

The major secondary metabolites and fatty acids occurring in the seeds of *Virola surinamensis* were monitored by GC-MS during germination and seedling development. The role as carbon source for seedling development was indicated considering that both classes of compounds were similarly consumed in the seeds and that no selective consumption of compounds could be detected.

## 1. Introduction

 Several neotropical trees produce fruits with large and heavy seeds [1]. *Virola surinamensis* is a myristicaceous tree growing in the Amazonian flooded plains and produces seeds during the rainy season [2, 3]. Seeds are viable shortly after ripening and are adapted to be dispersed by water or by large birds such as toucans and *araçaris*. The seedling formation can be divided in two distinct phases: seed germination and seedling development [4]. The cotyledons are hidden in the seed coat (cryptocotylar) and are storage organs of fatty material and polysaccharides that are recruited for the maintenance of seedling during its growth and development [5]. A study carried out on *V. venosa* revealed that the major lignans cubebin and dihydrokusunokinin accumulated in the seeds were not detected in its seedlings which accumulated a polyketide instead [6]. The major constituent identified in the seedling roots was shown to be the lignan sesamin, a minor constituent in the seeds. A different result were observed with *V. sebifera* in which a possible translocation of hydroxytetralone lignans and a preferential accumulation of a lignan hydroxy-otobain was observed in the whole seedlings [7].

 In view of the lack of systematic investigation regarding this important event in the reproduction of tropical trees, the translocation of secondary metabolites occurring in large seeds to be used as a defensive compounds in the seedlings remains as a hypothesis [8, 9].

**Figure 1:**
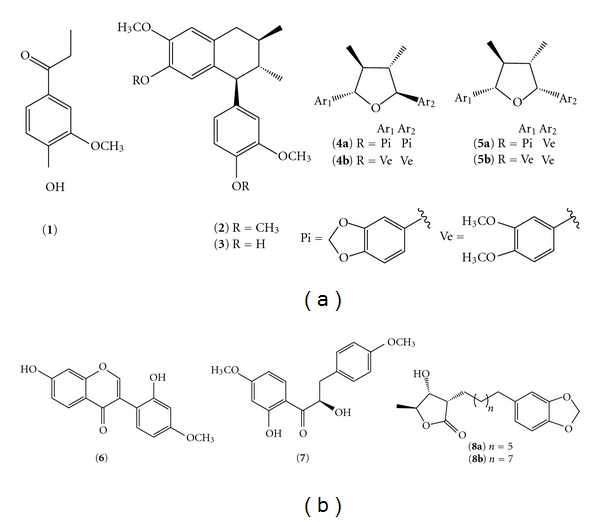
Chemical structures of the isolated substances: 4-hydroxy-3-methoxypropiophenone (**1**), galbulin (**2**), guaiacin (**3**), galbacin (**4a**), galbelgin (**4b**), calopeptin (**5a**), veraguensin (**5b**), 7,2′-dihydroxy-4′-methoxy-isoflavone (**6**), *α*,2′-dihydroxy-4,4′-dimethoxydihydrochalcone (**7**), juruenolide C (**8a**), and juruenolide D (**8b**).

*Virola surinamensis* seeds contain 15.4% of soluble tannins as a dry mass and the highest concentration of compounds with a probable defensive function yet recorded [10]. Their cotyledons are rich in triacylglycerols and free fatty acids. Phytochemical analysis of *V. surinamensis* seeds collected at Combu Island demonstrated the occurrence of lignoids, propiophenone, and *γ*-lactones in these organs [11]. Analysis of seedling leaves of *V. surinamensis* growing in the field, in greenhouse conditions and in micropropagated plantlets revealed the absence of lignans and the exclusive occurrence of juruenolide C (**8a**) (Figure 1) [12]. Herein, we wish to report the analyses of fatty acids and major secondary compounds in seeds of *V. surinamensis* in order to evaluate a selective consumption during the germination process.

## 2. Experimental Section

### 2.1. General Procedures

Preparative thin-layer chromatography (prep. TLC) was carried out on silica gel GF-254 (Merck) and column chromatography (CC) on silica gel 60H (0.005–0.045 mm) (Merck). The ^1^H NMR (200 MHz) and ^13^C NMR (50 MHz) spectra of samples were recorded on a Bruker-AC 200 in CDCl_3_ with tetramethylsilane (TMS) as an internal standard. EIMS was obtained at 70 eV on HP 5988-A.

### 2.2. Plant Material

Seeds of *Virola surinamensis* (Rol.) Warb. were collected in February 1995 at Combu Island (01°30′10′′S; 048°27′42′′W), near Belém, Pará State, Brazil. A dry voucher sample (LOPES-037) has been deposited in the SPF-Herbário do Instituto de Biociências da Universidade de São Paulo. Mature seeds were frozen for analysis or germinated as previously reported [13] and maintained at greenhouse facilities of Instituto de Química-USP.

### 2.3. Standards Isolation

One dried seed (320 mg), after the germination process, was extracted with CH_3_OH (3x 50 mL). The concentrated extract (70 mg) was suspended in CH_3_OH/H_2_O (6 : 4) and filtered through a Millipore membrane (0.45 *μ*m). The filtered extract was submitted to preparative on HPLC (RP-8, 10 *μ*m, 250 × 22 mm column; CH_3_OH/H_2_O 60 : 40 → CH_3_OH 100% (50 min), 8 mL·min^−1^, optimized conditions), followed by prep. TLC (silica gel; Hexane/EtOAc/*i*-PrOH or CH_2_Cl_2_/Me_2_CO) to yield 4-hydroxy-3-methoxypropiophenone (**1**, 1.6 mg) [14], galbulin (**2**, 5.5 mg) [15], guaiacin **3** (1.4 mg) [15], galbacin (**4a**, 2.0 mg) [16], galbelgin (**4b**, 1.0 mg) [17], calopeptin (**5a**, 1.6 mg) [18], veraguensin (**5b**, 5.0 mg) [19], 7,2′-dihydroxy-4′-methoxy-isoflavone (**6**, 1.5 mg) [20], *α*,2′-dihydroxy-4,4′-dimethoxydihydrochalcone (**7**, 1.8 mg) [21], juruenolide C (**8a**, 1.2 mg) [12], and juruenolide D (**8b**, 1.3 mg) [11]. All these compounds were identified by comparison of spectroscopic data with that reported in the literature.

### 2.4. Fatty Acids Analyses

Individual seeds before and after germination process were extracted (3x) with 200 mL of *n*-hexane. The transesterification of oils was carried out according to a procedure described by Maia and Rodrigues-Amaya, 1993 [22]. The methyl esters were dissolved with *n*-hexane (2 mg·mL^−1^), and 1 *μ*L was injected in a Hewlett-Packard 5890 gas chromatograph coupled to a Hewlett-Packard 5988 mass spectrometer in the condition previously described [12, 23].

### 2.5. Analyses of Secondary Compounds

Individual seeds, before and after the germination process, were extracted (3x) with 20 mL of CH_3_OH. The extract was concentrated to dryness and the residue dissolved with CH_2_Cl_2_ to obtain 2 mg·mL^−1^ as the final concentration, and 1 *μ*L was injected. All the analyses were performed with seven replicates in a Hewlett-Packard 5890 gas chromatograph coupled to a Hewlett-Packard 5988 mass spectrometer. The sample was injected (250°C) on a DB-5 column (30 m × 0.25 mm ID × 0.25 *μ*m of film tickness). The column temperature was initially 120°C (2 min), then programmed to 230°C at 7°C·min^−1^, kept at 230°C for 10 min, and then increased to 290°C in 15 min. The mass spectra were recorded at 70 eV. The identification of individual constituents was carried based on injection of isolated substances and comparison of their mass spectra.

### 2.6. Statistical Analysis

Statistical analyses were performed with the graphPad InStat software. All values were reported as means ± SEM, and were analyzed for statistical significance by two way analysis of variance followed by Student test. The minimum level of significance considered was *P* < 0.05.

## 3. Results and Discussion

 Two groups of seeds of *V. surinamensis*, before germination (BG) and 6-7 months after germination (AG), were analyzed for fatty acids and major secondary metabolites. The second group (AG) showed a decrease of 30% in dry weight, but without significant changes in the extraction yield (Table 1). These results are in agreement with Durian's hypothesis, in which seeds are a nutrient storage organ to supply the seedling during the growth process [8]. The analyses of fatty acids content carried out in seeds of *V. surinamensis* before and after germination showed similar relative content of lauric acid (16%), myristic acid (70%), palmitic acid (6%), and stearic acid (8%). This result is similar to that previously reported [23], and since no preferential uptake of fatty acids could be detected, the major role of fatty material as carbon source is clearly supported (Table 2).

**Figure 2:**
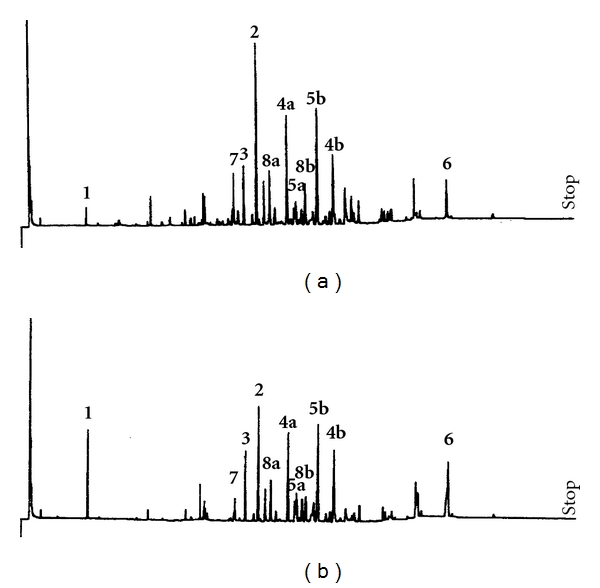
GC profile of secondary metabolites before (a) and after (b) germination of *V. surinamensis* seeds.

 The secondary metabolites in both groups of seeds of *V. surinamensis* were analyzed by GC-MS. The chromatographic profile observed for both groups exhibited the predominance of galbulin (**2**), galbacin (**4a**), and veraguensin (**5b**) as the major compounds (Figure 2). After statistical analyses, no significant variation was observed in the relative content of monitored compounds, except to compound **1** (*P* < 0.05) (Table 2).

 From *V. surinamensis,* new substances were isolated [24] and some neolignans showed allelopathic properties [25]. Recently, other neolignans showed antiinflammatory and antileishmanial activities [26, 27]. In addition, the increase of phenolic compounds was observed after elevated CO_2_ submission in *V. surinamensis *[28] and a strong inhibition of CO_2_ assimilation by sun exposure [29]. However, the analyses of the composition occurring in the seeds of this species during germination and seedling processes had not been studied yet.

 In summary, the germination of *V. surinamensis* seeds and the seedling development are processes in which both fatty acids and secondary metabolites (lignans, isoflavonoids, and juruenolides) are equally consumed in the seeds indicating their physiological role as energy and carbon source, or by other physiological function. In spite of the large concentration of lignans in the seeds (8.5% as dry weight basis), no specific translocation to the seedlings and no consumption of a specific compound from the seeds could be detected. The lignans could have biological importance to the seeds, but after the lignans uptake to the seedling, our results, in addition to the previous phytochemical investigations [12], reinforce the use of these compounds as energy and carbon source by the seedlings.

## Figures and Tables

**Table 1:** Arithmetic mean of dry weight extracts and yields of *V. surinamensis* seeds.

Seeds*	Dry weight (mg)	Extract (mg)	Yield (%)
BG (*n*-hexane)	1030	310	30
AG (*n*-hexane)	290	81	28
BG (CH_3_OH)	950	180	19
AG (CH_3_OH)	270	46	17

BG: seeds before germination; AG: seeds after germination.

*Number of seeds used in each experiment = 7.

**Table 2:** Relative contents of secondary metabolites and fatty acids in *V. surinamensis* seeds.

	Seeds before germination			Seeds after germination			Statistical analysis
	S	SEM	CI	S	SEM	CI	*P*
**1**	2.01	0.461	0.729–3.291	0.64	0.319	0.0–1.528	0.041 (s)
**2**	14.25	1.800	9.179–19.321	13.37	1.766	8.974–18.766	0.884 (ns)
**3**	3.92	0.449	2.672–5.168	3.53	0.299	2.696–4.360	0.488 (ns)
**4a**	10.02	0.937	7.417–12.623	9.96	1.161	6.735–13.181	0.958 (ns)
**4b**	4.05	0.821	1.772–6.328	3.50	0.626	1.757–5.235	0.606 (ns)
**5a**	2.93	0.439	1.708–4.148	4.40	0.853	2.036–6.772	0.162 (ns)
**5b**	9.69	1.336	5.983–13.401	11.30	1.454	7.260–15.336	0.439 (ns)
**6**	3.58	0.562	2.015–5.141	2.28	0.2448	1.601–2.960	0.067 (ns)
**7**	2.28	0.292	1.471–3.197	2.06	0.264	1.333–2.799	0.595 (ns)
**8a**	3.71	0.361	2.707–4.709	4.08	0.219	3.472–4.962	0.402 (ns)
**8b**	1.98	0.157	1.552–2.424	2.00	0.365	0.995-0.021	0.961 (ns)
**L**	15.95	0.331	14.820–17.340	16.08	0.453	14.820–17.340	0.821 (ns)
**M**	71.04	1.230	67.630–74.450	69.78	0.491	68.420–71.130	0.356 (ns)
**P**	6.04	0.480	4.690–7.380	6.00	0.090	5.740–0.260	0.945 (ns)
**S**	7.92	0.040	7.820–8.020	7.92	0.248	7.230–0.610	0.438 (ns)

S: mean; SEM: standard error mean; CI: confidence interval (95%); *P* < 0.05; s: statistically significant; ns: not statistically significant; **L**: lauric acid; **M**: myristic acid; **P**: palmitic acid; **S**: stearic acid.
